# The Social Construction of Stigma in Aged-Care Work: Implications for Health Professionals’ Work Intentions

**DOI:** 10.1093/geront/gnac002

**Published:** 2022-01-08

**Authors:** Asmita V Manchha, Kïrsten A Way, Ken Tann, Michael Thai

**Affiliations:** School of Psychology, The University of Queensland, Brisbane, Queensland, Australia; School of Psychology, The University of Queensland, Brisbane, Queensland, Australia; The University of Queensland Business School, Brisbane, Queensland, Australia; School of Psychology, The University of Queensland, Brisbane, Queensland, Australia

**Keywords:** Aged-care workforce, Dirty work, Institutional care, Recruitment, Stigma

## Abstract

**Background and Objectives:**

Although society has cultivated a deeper appreciation for essential health services, societal discourses reinforce a stigma of working in aged care. Drawing on dirty work and Stigma Theory, this study aims to investigate stigma in the context of recruiting health professionals.

**Research Design and Methods:**

We employed a mixed-methods design to examine the nature and implications of the stigma of working in aged care. A path analysis was used to test whether health professionals’ (*n* = 159) negative perceptions of aged-care work would negatively predict their willingness to work in aged care. A linguistic analysis was conducted to understand how health professionals’ (*n* = 168) use of language positions themselves toward or away from engaging in aged-care work.

**Results:**

Quantitative findings revealed that perceptions of physical taint directly predicted lower willingness to perform aged-care work. Perceptions of social taint, moral taint, and poor occupational conditions negatively predicted willingness to work in institutional aged care, indirectly via social devaluation. Findings from the linguistic analysis demonstrated that health professionals (re)produce stigma through aligning themselves with devaluing discourses about aged-care workers, work, and institutions.

**Discussion and Implications:**

This study provides insight about the role that stigma plays in the aged-care recruitment crisis, with implications for aged-care institutions. Societal discourse may obstruct the employment of health professionals in aged care because it can (re)produce the stigma of working in aged care. Recommendations for ways to reduce the impact of this stigma include public messaging and training.

Social discourses throughout the coronavirus pandemic have foregrounded the value of health professionals in the fight against coronavirus disease 2019 (COVID-19; Daly et al., 2020), with the media fostering a “superhero narrative” when reporting the experiences of health professionals in emergency departments (McAllister, Lee Brien et al., 2020). These discourses, in addition to promoting a deeper appreciation for health workers on a global scale, may also assist in recruiting workers by positioning this work as important and socially desirable. For example, the call out for medical students to fill labor shortages has been compared to rhetoric associated with enlisting soldiers in world wars (Thompson & Lovegrove, 2020).

On the flip side, social discourses may also hinder the recruitment of essential workers in low-wage and direct care work (Scales, 2021) like aged care (Clarke & Ravenswood, 2019; Henderson et al., 2008; Manchha et al., 2021). The chronic understaffing in aged care is a current global concern, as the demand for aged care is disproportionate to the quantity of available qualified workers. It is predicted that 1.3 million additional jobs within this sector will need to be filled between 2018 and 2028 in the United States alone (Scales, 2021).

Despite providing an essential health service throughout the pandemic, the aged-care sector has been socially devalued in comparison to acute care (Monahan et al., 2020). Care workers were often overlooked in health campaigns like “the clap for medical staff” (McAllister, Ryan et al., 2020). Furthermore, the aged-care workforce experienced first-hand their marginalized position when they were required to work without access to personal protective equipment even 2 months into COVID-19 (McGilton et al., 2020). We argue that the pandemic has reinforced an aged-care stigma: “an attribute that is deeply discrediting and reduces the worth of an individual in other people’s eyes” (Goffman, 1963, p. 3). Specifically, social discourses have highlighted problems in this occupation (e.g., low pay, hazardous working conditions, overbearing workloads; Scales, 2021).

We explore this occupational stigma from the perspective of health professionals to determine whether it has negative ramifications for the perceived value of working in aged care and, subsequently, could further discourage workers from engaging in aged-care work. We refer to health professionals as groups (i.e., allied health, nurses, social assistance, doctors) who do not form a part of the aged-care workforce because they currently provide health care in other specializations/settings. In this mixed-methods study, we first empirically test the relationship between health professionals’ perceptions of taint, conditions, and social devaluation of aged-care work, as well as their willingness to work in aged care contexts (Figure 1).

**Figure 1.**
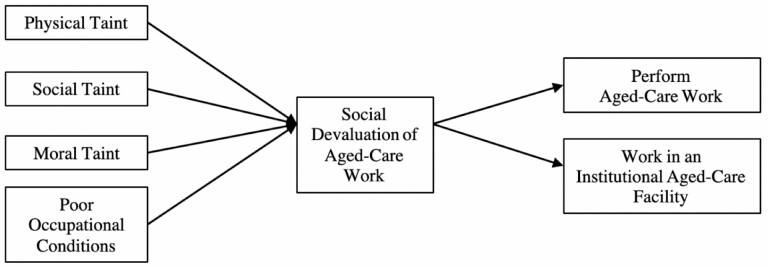
Conceptual model of negative perceptions of aged-care work on willingness to work in aged care through social devaluation of aged-care work.

We further employ a form of linguistic analysis known as Appraisal (Martin & White, 2005) to investigate health professionals’ and society’s discourses, and how their use of language orients them and others toward aged-care work during COVID-19. We do this to gain insight into the social processes underlying the stigma of working in aged care and extend existing knowledge about the impact of stigma on the recruitment of workers in aged care.

We apply a theoretical framework from the dirty work literature (Ashforth & Kreiner, 2014; Hughes, 1958) alongside Stigma Theory (Link & Phelan, 2001; Yang et al., 2007) to answer three research questions: (a) “Do health professionals’ perceptions of aged-care work negatively predict their willingness to work in aged care?”; (b) “What social mechanisms might be at play that helps explain these relationships?”; and (c) “How do health professionals position themselves toward social discourses about the devaluation of aged care during COVID-19?” Whereas the dirty work literature (Ashforth & Kreiner, 2014) describes the different forms of tainted work (i.e., physical, social, moral) that may be associated with stigmatized occupations, it does not explicate the detailed mechanisms through which stigma and its negative consequences manifest. We therefore integrate Stigma Theory (Link & Phelan, 2001; Yang et al., 2007), which proposes that negative social evaluations can lead to status loss and discrimination against stigmatized entities, to help explain how negative social evaluations can affect workers’ willingness to engage with aged-care work.

## Health Professionals’ Negative Perceptions of Aged Care

We build on existing literature about the low value placed on aged-care work (Manchha et al., 2021) by empirically testing the factors that may predict the devaluation of aged-care work among health professionals. In line with the dirty work literature (Ashforth & Kreiner, 2014), which primarily examines occupations in terms of taint and low occupational status, we theorize that perceptions of taint and poor occupational conditions may lead health professionals to evaluate aged care as a socially devalued occupation. Aged care in Western cultures has historically been associated with physical taint (as it can involve exposure to bodily fluids and death) and social taint (because it provides a service to a devalued societal group, e.g., older adults, and a servile relationship; Hughes, 1958; Ostaszkiewicz et al., 2016). Furthermore, we also examine moral taint, which has been overlooked in past theorizing around the stigma and devaluation of aged-care work. Moral taint can apply to unethical work that defies social norms (Ashforth & Kreiner, 2014). We argue that the prevalence of narratives surrounding unethical aged care (e.g., elder abuse and neglect) and the marketization of aged care may tarnish the virtuous nature of aged-care work, thus producing moral taint associated with working in aged-care institutions (Timonen & Lolich, 2019). Perceptions of poor occupational conditions (i.e., low pay, high workloads) further frame aged care as an unattractive career and perpetuate that this occupation is culturally devalued (Ashforth & Kreiner, 2014).

Based on this theorizing, we make the following hypothesis:

H1 Perceptions of (a) physical taint, (b) social taint, (c) moral taint, and (d) poor occupational conditions associated with aged-care work will positively predict social devaluation of aged care.

We further theorize that health professionals may be less willing to work in aged care because they could incur stigma from performing or being associated with a socially devalued occupation. Willingness to perform aged-care work is defined as favorable intentions to deliver care services for recipients primarily aged 65 and older (e.g., bathing, distributing medication), whereas willingness to work in an institutional aged-care facility is defined as favorable intentions to work in a formal institutional aged-care context. Past studies have found health professionals report less interest in performing aged-care work based on perceptions that aged care is less valued because it involves basic care work rather than advanced medical skills (Neville, 2016). Similarly, devaluing discourses about institutional aged care (facilities being depressing, ill-equipped, and primarily catering for residents with higher care needs; Roth et al., 2016) may deter health professionals from wanting to work in this setting. Thus, we hypothesize:

H2(a) Perceptions of social devaluation of aged-care work will negatively predict willingness to perform aged-care work.

H2(b) Perceptions of social devaluation of aged-care work will negatively predict willingness to work in an institutional aged-care facility.

Building on these proposed direct effects, we integrate the dirty work literature with Stigma Theory (Link & Phelan, 2001) to posit social devaluation as a mechanism through which occupational taints and poor occupational conditions predict willingness to perform aged-care work and work in aged-care institutions. This mechanism can be understood via social processes from Stigma Theory (Link & Phelan, 2001), wherein groups that become associated with negative characteristics experience status loss. We suggest that, due to the different types of taint and negative occupational conditions that they perceive aged-care work is associated with (i.e., negative attributes), health professionals may want to distance themselves from engaging in devalued aged-care work (i.e., to avoid status loss) and be less inclined to perform aged-care work or work in an institutional aged-care facility (i.e., discriminate against aged-care work).

H3(a) Perceptions of taint (physical, social, moral) and poor occupational conditions will have an indirect effect on willingness to perform aged-care work through perceptions of social devaluation of aged care work.

H3(b) Perceptions of taint (physical, social, moral) and poor occupational conditions will have an indirect effect on willingness to work in an institutional aged-care facility through perceptions of social devaluation of aged-care work.

## Role of Societal Discourses About Aged Care in the Context of COVID-19

Health professionals’ willingness to work in aged care may be influenced by how they interpret social discourses about aged care within a situational context. We posit that health professionals may internalize discourses about aged care during COVID-19 in terms of stigma, which is often exacerbated under a crisis (Pervan & Bove, 2015). For example, COVID-19 adds a contagion risk to the already demanding work performed by aged-care workers (Blanco-Donoso et al., 2021). Grounded in Yang et al.’s (2007) interpretation of Stigma Theory (Link & Phelan, 2001), societal discourses may manifest in terms of stigma through an intersubjective process. This process refers to the social construction of stigma via interpersonal communication (i.e., exchange of language and meanings) between the stigmatizer and stigmatized (Yang et al., 2007, p. 1532). Based on this theorizing, communication between health professionals and society may persuade health professionals to align with discourses about the devaluation of aged care and interact with aged care in ways that (re)produce stigmatization (i.e., unwillingness to work in aged care). We aim to identify patterns in the language health professionals use to engage with these discourses that devaluate aged care.

## Method

### Participants and Procedure

Health professionals (*N* = 168) were recruited via Academic Prolific to complete a two-part online survey. A total of 159 (see Author Note 1) participants (107 females, 52 males; *M*_age_ = 36.94, *SD* = 10.90; age range = 20–65 years) currently working in roles outside of the aged-care sector were included in the study (allied health [46.5%], nursing [27.7%], social assistance [13.2%], or physician [12.6%]). Nearly two thirds (64.2%) of participants reported that they previously worked in the aged-care sector prior to their current position. Participants were from the United Kingdom (61%) and the United States (39%).

At Time 1, participants were asked to report their perceptions (see Author Note 2) of (a) taint (physical, social, moral), (b) poor occupational conditions, and (c) social devaluation of aged-care work. At Time 2, 1 week later, participants were asked to report their (d) willingness to perform aged-care work/work in an institutional aged-care facility and (e) demographic questions. Participants were also invited at Time 1 to write a short response to an open-ended question: “How has COVID-19 impacted your perceptions of working in aged care?” We were not interested in how participants felt about COVID-19, rather, how they would position themselves in alignment (or disalignment) with stigmatizing discourses about aged-care work made more evident during the pandemic, without directly asking them about “stigma.” We gave them this prompt as a way to do that. This study was granted ethics approval by the Human Research Ethics Committee of The University of Queensland.

#### Physical taint

A three-item measure comprised of items adapted from Bickmeier (2018) assessed the extent to which participants associate physical taint with aged-care work (e.g., “Most people in society tend to think aged-care work is unclean”; α = 0.93).

#### Social taint

A four-item measure comprised of items adapted from Banks (2018) assessed the extent to which participants associate social taint with aged-care work (e.g., “Most people in society tend to think aged-care work involves caring for people who are less important”; α = 0.85).

#### Moral taint

A three-item measure comprised of items adapted from Lai et al. (2013) and Valtorta et al. (2019) assessed the extent to which participants associate moral taint with aged-care work (e.g., “Most people in society tend to think aged-care work is unethical”; α = 0.82).

#### Poor occupational conditions

A five-item measure comprised of items adapted from Chou et al. (2002) assessed the extent to which participants associate poor occupational conditions with aged care (e.g., “Most people in society tend to think aged care involves unsatisfactory pay and remuneration”; α = 0.89).

#### Social devaluation of aged-care work

A five-item measure comprised of items adapted from Neville (2016) assessed the extent to which participants associate social devaluation with aged-care work (e.g., “Most people in society tend to think working in aged care is devalued”; α = 0.92).

All of the scales above used a 7-point Likert scale with response options of 1 “strongly disagree” to 7 “strongly agree.”

#### Willingness to perform aged-care work

A two-item measure comprised of items adapted from Evans-Lacko et al. (2011) assessed participants’ willingness to perform aged-care work (e.g., “In the future, would you be willing to perform aged-care work?”; α = 0.90).

#### Willingness to work in an institutional aged-care facility

A two-item measure comprised of items adapted from Evans-Lacko et al. (2011) assessed participants’ willingness to work in an institutional aged-care facility (e.g., “In the future, would you be willing to work in an institutional aged-care facility?”; α = 0.93).

The two scales above used a 5-point Likert scale with response options of 1 “definitely not” to 5 “definitely yes.”

### Analysis

#### Quantitative

We conducted a series of path analyses using a software package (AMOS version 25) to examine the direct and indirect effects between hypothesized predictors and consequences. We employed the bias-corrected bootstrapping method (Fritz & MacKinnon, 2007) to test indirect effects.

#### Linguistic analysis

**Figure 2.**
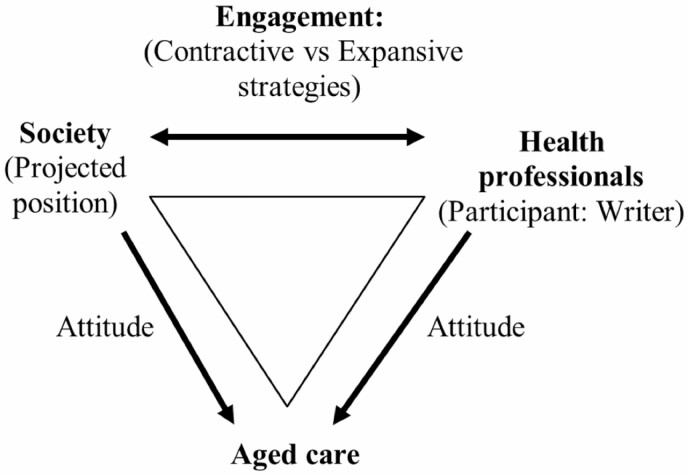
Linguistic model.

We employed linguistic tools (i.e., Appraisal framework; Martin & White, 2005) developed within Systemic Functional Linguistics to investigate how health professionals position themselves toward societal discourses about aged care during COVID-19 (Figure 2), in their written responses to an open-ended survey question. Within the Appraisal framework, the engagement system (Martin & White, 2005, p. 122) provides a rigorous and principled method for identifying a writer’s position through analyzing their language choices at a microscopic level. These choices align or disalign a writer with respect to a particular position that they project as the norm (or the way society perceives it should be; e.g., the devaluation of aged care). We examined the underlying patterns of how aged care is being framed in language across occupations rather than individual expressions of participants. This enabled us to generate insight about the process through which stigma is made socially meaningful in communication.

First, we screened health professionals’ (*N* = 168) written responses to check whether or not they responded to the question (i.e., whether they actually wrote about perceptions of aged care). All participants’ responses addressed the question and were thus retained. Responses varied in length from a few words to a page of text. Second, the first author coded the textual responses according to the following Appraisal categories provided in the work of Martin and White (2005): (a) attitude types (affect, judgment, appreciation), (b) attributed sources (health professional, society, media), and (c) target of attitudes (aged-care workers, aged-care work, aged-care institutions). These categories provide a systematic way to describe recurring patterns in language use. Affect refers to expressions associated with emotional reactions, judgment refers to expressions evaluating a person’s character (e.g., aged-care workers) based on ethics/moral sensibilities, while appreciation refers to expressions ascribing value to objects and situations (e.g., institutions; Martin & White, 2005). We reviewed the responses with two groups of researchers to ensure the meaning of the sentences was interpreted correctly. Third, we identified recurring attitudinal patterns (i.e., combinations of attitude types and targets) based on predefined Appraisal categories with explicit descriptors (Martin & White, 2005). We classified these statements using the engagement system to identify the writer/respondent’s positioning and dis/alignment with the projected position (i.e., discourses that are generally expected; devaluing aged care).

## Quantitative Results

Means, standard deviations, and bivariate correlations between the variables of interest are presented in Table 1.

**Table 1.** Means, Standard Deviations, and Zero-Order Correlations Among the Hypothesized Predictors

Variable	*M*	*SD*	1	2	3	4	5	6
1. Physical taint	3.56	1.56	—					
2. Social taint	3.42	1.41	0.53**	—				
3. Moral taint	2.76	1.21	0.41**	0.39**	—			
4. Poor occupational conditions	5.00	1.20	0.24*	0.43**	0.28**	—		
5. Social devaluation of aged-care work	4.15	1.16	0.38**	0.64**	0.40**	0.54**	—	
6. Willingness to perform aged-care work	3.48	1.07	−0.18*	−0.06	−0.15	−0.11	−0.09	—
7. Willingness to work in an institutional aged-care facility	3.06	1.12	−0.02	−0.12	−0.11	−0.14	−0.18*	0.70**

*p < .05, **p < .01.

**Figure 3.**
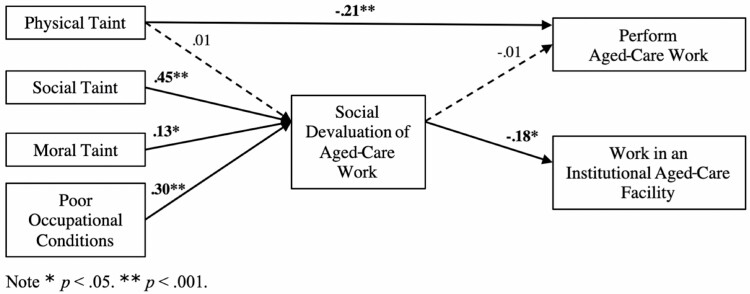
Tested model of perceptions of aged-care work on willingness to work in aged care through social devaluation of aged-care work.

The initial fit of the path model, as informed by the conceptual model (Figure 1), was poor, χ ^2^(15) = 245.01, *p* < .001, χ ^2^/df ratio = 16.33, Tucker-Lewis Index (TLI) = 0.34, Comparative Fit Index (CFI) = 0.33, Root Mean Square Error of Approximation (RMSEA) = 0.31 (0.28, 0.35), Standardized Root Mean Residual (SRMR) = 0.25. Inspection of the modification indices revealed a direct path between physical taint and willingness to perform aged-care work that could be freed to improve model fit (β = −0.21, *p* < .001). Given that perceptions of physical taint that apply to aged care may also apply to health care generally (i.e., working with bodily fluids and potential exposure to death), it was theoretically viable that the relationship between physical taint and a person’s willingness to engage in aged-care work generally (e.g., treating an older patient in hospital) was not mediated by negative social evaluations of aged care per se. As such, we deemed it acceptable to free this path. Freeing a number of covariances and the direct path outlined above improved model fit considerably. The final model (Figure 3) fit the data well, χ ^2^(7) = 4.68, *p* = .70, χ ^2^/df ratio = 0.70, TLI = 1.00, CFI = 1.00, RMSEA = 0.00 (0.00, 0.08), SRMR = 0.02. We also tested for differences between professional roles (Supplementary Material).

### Direct Effects

Hypothesis 1(a) was not supported as physical taint did not predict social devaluation (β = 0.01, *p* = .86). However, we found support for hypothesis 1(b)–1(d) with social taint (β = 0.45, *p* < .001), moral taint (β = 0.13, *p* < .05), and poor occupational conditions (β = 0.30, *p* < .001) predicting greater perceived social devaluation. Hypothesis 2(a) was not supported as there was a nonsignificant relationship between social devaluation and willingness to perform aged-care work (β = −0.01, *p* = .90). Supporting hypothesis 2(b), however, social devaluation was a negative predictor of willingness to work in an institutional aged-care facility (β = −0.18, *p* < .05).

### Indirect Effects

While we did not find support for indirect effects of taints and poor occupational conditions on willingness to perform aged-care work, we did find that the standardized indirect effects of social taint, moral taint, and poor occupational conditions on willingness to work in an institutional aged-care facility through social devaluation were significant (Table 2), partially supporting hypothesis 3(b).

**Table 2.** Indirect Effects of Negative Perceptions of Aged-Care Work on Willingness to Work in Aged Care

Negative perceptions of aged care work	Willingness to perform aged-care work	Willingness to work in an institutional facility
Physical taint → Social devaluation	0.01 (0.01); [−0.02, 0.01]	−0.01 (0.01); [−0.04, 0.02]
Social taint → Social devaluation	−0.01 (0.04); [−0.08, 0.08]	−**0.08 (0.04); [**−**0.18,** −**0.01]**
Moral taint → Social devaluation	−0.01 (0.01); [−0.03, 0.02]	−**0.02 (0.02); [**−**0.07,** −**0.001]**
Poor occupational conditions → Social devaluation	−0.01 (0.03); [−0.06, 0.05]	−**0.05 (0.03); [** −**0.12,** −**0.004]**

*Notes:* Standard errors are listed in parentheses and confidence intervals (CIs) in square brackets. All coefficients are standardized, bootstrapped 95% CI. Significant effects appear in bold type.

## Linguistic Results

We identified how health professionals dis/aligned themselves with social discourses about aged care during COVID-19, through examining how they position themselves (e.g., contractive vs. expansive) in terms of dialogic engagement (Martin & White, 2005). For example, they may either use a contractive strategy to constrain alternative positions toward aged care or an expansive strategy to include alternative positions. These positions may involve a positive or negative attitudinal statement with respect to an attitude type (i.e., affect, judgment, appreciation) and its target (i.e., aged-care workers, work, institutions) that align or disalign with their perception of the (projected) societal position. We define negative attitudinal statements as those aligning with the devaluation of aged care and positive attitudinal statements as those disaligning with the devaluation of aged care. Table 3 presents a summary of positioning strategies and example attitudinal statements about aged care.

**Table 3.** Summary of Positioning Strategies and Example Attitudinal Statements About Aged Care

Strategy	Attitude	Representative quotes	Target	Source
**Alignment with the devaluation of aged care**				
*Contractive strategy*	Negative valuation***	I personally think that the *vast majority* of aged-care workers do care [+tenacity] *but* [counter] are *desperately* undervalued [−valuation] by society. (*Participant 98: Allied Health*)	Workers	Health professionals
		*I’ve worked in that field before moving to surgical nursing* [pronounce] it’s work that is underpaid [−valuation] and undervalued [−valuation]. (*Participant 42: Nurse*)	Institution	Health professionals
		*The media’s portrayal of working in aged care* [endorse] has stigmatized [−valuation] and belittled [−valuation] the work. (*Participant 69: Social Assistance*)	Work	Media
		If you work in a nursing facility, *the media has portrayed it* [endorse] as a dangerous [−valuation] place. (*Participant 41: Allied Health*)	Institution	
	Negative tenacity**	*The media has portrayed that* [endorse] aged care companies and institutions don’t look out for their employees [−tenacity] and don’t put their residents or employees first [−tenacity]. (*Participant 136: Allied Health*)		
*Expansive strategy*	Negative valuation***	Made work *seem* [entertain] unsafe [−valuation] and dangerous [−valuation]. (*Participant 21: Doctor*)	Work	Health professionals
		*It seems* [entertain] like a scary [−reaction] time to be working in aged care. (*Participant 10: Allied Health*)	Institution	
		*I feel like* [entertain] workers have been abandoned [−valuation] by the government and by society. (*Participant 142: Allied Health*)	Workers	Health professionals
	Negative normality**	*I believe* [entertain] workers have been *very* unfairly [−normality] treated. (*Participant 141: Allied Health*)		
	Negative security*	*I would* [entertain] feel *more* afraid [−security] to work in aged care *now*. (*Participant 25: Social Assistance*)	Institution	
**Disalignment with the devaluation of aged care**				
*Contractive and expansive strategy*	Positive valuation***	*I see* [entertain] *now* how undervalued [−valuation] we are as workers by the general public, *but* [counter] how *highly* respected [+valuation] we’ve become to *families of the aged we care for* [attribute]. (*Participant 63: Allied Health*)	Workers	Health professionals
	Positive tenacity**	There is a *usually* unspoken feeling that aged- care workers are responsible for transmission of virus ... [−tenacity] *while on the other side* [counter] *there have been examples of* [attribute] care *above and beyond* the call of duty [+tenacity]. (*Participant 91: Doctor*)		

*Note:* Attitude types: *Affect, **Judgment, and ***Appreciation.

### Alignment with the Devaluation of Aged Care: Contractive Strategies

Some health professionals used contractive strategies to frame their alignment with the devaluation of aged care, which involved rejecting alternative positions, reinforcing or endorsing the projected position. One way that participants construed their rejection of alternative positions was by acknowledging alternative positions only to *counter* these views using expressions like “*but*”. For example, “there are wonderful caring people in aged care *but* they are underappreciated and underpaid” (Participant 38: Social Assistance). This enabled participants to dismiss positive attitudes and reinforce negative attitudes about aged-care workers. Participants also demonstrated their explicit support for the devaluation of aged care by personally aligning themselves with negative attitudinal statements. Health professionals *pronounced* (see Author Note 3) themselves as a credible source to show their motivation to align with the devaluation of aged care arose from a personal experience (i.e., “*As I have an insight into the profession*, I already know that the industry is massively underfunded and the pressures of COVID-19 have only highlighted this” [Participant 93: Nurse]).

Additionally, we found some health professionals framed the media as an external authorial voice to *endorse* their negative attitudes about aged-care work and institutions (i.e., “*the media thinks* aged care workers don’t care for their patients and would rather let their patients die from COVID-19 than care for them” [Participant 52: Doctor]). Primarily, these attitudes were construed in terms of valuation, which refers to perceptions about what society deems as worthy and how people value them (Martin & White, 2005). Specifically, the media reported negative valuation (i.e., “low status”) about working in aged care.

The media also *endorsed* negative judgment about the character of people working in aged-care institutions in relation to their tenacity (i.e., whether a person is considered resolute and dependable, see Martin & White, 2005). People who work in aged-care facilities were framed as careless (i.e., negative tenacity), “facilities *do not care* about their patients” (Participant 71: Allied Health), the use of the negative invokes the cultural expectation that aged-care institutions should be considerate of their clients.

### Alignment with the Devaluation of Aged Care: Expansive Strategies

Alternatively, health professionals may expand the scope of alternative positions to recognize multiple views in society. We found many participants extended their repertoire of attitudinal statements about the negative valuation beyond aged-care workers and institutions, by attributing negative valuation with another target—aged-care work. Additionally, a few participants framed attitudinal statements about institutions in terms of a subtype of appreciation—reaction, which evaluates the value of an object or situation in terms of affective responses (Martin & White, 2005). These participants consistently articulated negative reactions toward aged-care institutions in relation to its poor working conditions. Although health professionals can *entertain* additional positions (e.g., devaluation of aged-care work and negative reactions toward institutions), they may negotiate their positioning to others using language like “*it seems*” and “*presume it is*” as a way of endorsing majority views without explicitly rejecting certain positions. For example, “*it seems* to be fraught with risk during a pandemic” (Participant 100: Nurse) may invite their respondent to agree or disagree. Participants can then frame their response to either open up or shut down alternative positions based on whether it reflects the majority’s view.

In contrast, a majority of health professionals aligned with discourses about the devaluation of aged care through acknowledging negative attitudinal statements about aged-care workers and institutions. Participants used personal values such as “*I believe*” to closely position themselves toward the devaluation of aged care. For example, “*I believe* carers in care homes receive very poor money for difficult work” (Participant 40: Nurse). Specifically, participants framed negative attitudinal statements about workers in terms of valuation and a subtype of judgment—normality, which refers to evaluating a person based on how distinguished they are compared to others. An individual’s character can be viewed in terms of positive normality (e.g., celebrated) or negative normality (e.g., unfortunate; Martin & White, 2005). Participants frequently positioned people who work in aged care as an ill-fated group (negative normality). Participants also construed negative attitudinal statements about aged-care institutions in relation to security, a subtype of affect that focuses on evaluating emotional reactions associated with anxiety (Martin & White, 2005). Feelings of uneasiness (e.g., “*afraid*,” “*scared*”) were associated with working in institutions throughout COVID-19. Overall, some health professionals may have entertained alternative attitudinal statements; however, these additional positions were all aligned to the projected position—the devaluation of aged care.

### Disalignment with the Devaluation of Aged Care: Contractive and Expansive Strategies

A minority of health professionals in our sample construed their disagreement with the devaluation of aged care. These participants framed positive attitudinal statements about aged-care workers in terms of valuation and tenacity, using contractive strategies. For example, the participant would initially acknowledge a position they perceived was generally expected (i.e., the devaluation of aged care) and then would reject this perspective through expressing a favorable opinion about aged-care workers, “it is a more challenging time than ever *but* more valuable at the same time” (Participant 30: Doctor). We recognized participants also used expansive strategies (e.g., entertain) to articulate the projected position but then would externalize themselves from their alternative position. Participants would *attribute* the alternative position to an external voice such as, “families of the aged we care for” or an unidentified source “there have been examples ….” As a result, some health professionals would distance themselves from attitudinal statements, which may enable them to adjust their position according to whether their respondent aligns/disaligns with this alternative position. Consequently, these health professionals’ who disalign with the devaluation of aged care may actually be unsuccessful at challenging this stigma (i.e., advocating) for the alternative position.

## Discussion

This study investigated health professionals’ negative perceptions of aged-care work and societal discourses about the devaluation of aged care. Our findings suggest that perceptions of social taint, moral taint, and poor occupational conditions had a negative indirect effect on willingness to work in an institutional aged-care facility via social devaluation. That is, health professionals who perceived that aged-care work was socially/morally tainted and associated with poor occupational conditions were also more likely to believe that aged-care work was devalued by society and, in turn, were less willing to work in an institutional aged-care facility. Drawing on Stigma Theory (Yang et al., 2007), we recognize that health professionals may refuse to work in institutional aged care in order to distance themselves from the social devaluation attributed to aged-care institutions and any association with such facilities which may reflect negatively on themselves. On the other hand, we found that they perceive performing aged-care work itself as distinct from working in an aged-care facility and may not associate it with social devaluation.

Despite finding no indirect association of perceptions of physical taint on willingness to work in aged care or aged care institutions through social devaluation, we found that health professionals’ perceptions of physical taint directly predicted lower willingness to perform aged-care work. This finding aligns with the existing dirty work literature in aged care, which argues that people are discouraged to engage in what they perceive to be “foul and disgusting” work (Clarke & Ravenswood, 2019). This is a new and interesting finding, as it reveals health professionals are reluctant to perform aged-care work based on its inherent physically tainted nature, regardless of whether this work is perceived as devalued. It also indicates that the “dirty” nature of aged-care work is not a key driver of why aged-care work is devalued, at least in the eyes of health professionals. We argue that health professionals may have normalized that physical taint is a component of working in health care, thus perceiving physical taint is not associated with the value attributed to a health occupation.

We further examined the contextual nuances of this stigma through a linguistic analysis. Specifically, we investigated how health professionals engage with social discourses about aged care in the context of COVID-19. Our findings demonstrated that health professionals predominantly align themselves with societal discourses of the devaluation of aged care, which supports an established body of literature that positions aged care negatively (Banks, 2018; McGilton et al., 2020). We found, regardless of whether they employ contractive (i.e., suppress multiple voices) or expansive (i.e., represent multiple voices) engagement strategies, health professionals’ language choices suggested alignment with the societal devaluation of aged care. We identified participants who disaligned themselves with the devaluation of aged care were unlikely to challenge this established position. Health professionals externalized themselves from the alternative position, which hindered their capability to challenge the dominant discourse. We infer health professionals may distance themselves from the alternative position because of the interpersonal cost associated with rejecting the projected position of devaluation (e.g., scrutiny, marginalization). Alternatively, these participants may lack the linguistic resources to successfully challenge the devaluation of aged care. For example, participants may not be able to articulate alternative views.

We also discovered that health professionals draw explicitly on external sources to endorse specific positions. In particular, participants proclaimed their perspective toward the devaluation of aged care was attributed to the media. Through analyzing language choices, we found “*the media*” was personally identified (compared to generic sources like “*they*,” “*society*”) as an institution that endorsed health professionals’ negative attitudinal statements. We inferred that participants perceived the media as an authoritative source, through observing that this external voice was strongly assimilated in the text. For example, the participant reinforced their position by referring back to what had been said by the media previously. Thus, we suggest the media’s endorsement of negative attitudinal statements may reassure health professionals that their shared positioning (and alignment with the devaluation of aged care) is socially accepted and normative.

Findings from both quantitative and linguistic analyses revealed that health professionals reported stronger negative attitudes toward aged-care institutions in comparison to aged-care work. In line with the dirty work literature (Ashforth & Kreiner, 2014) and Stigma Theory (Yang et al., 2007), we suggest that perceptions of occupational taint, poor occupational conditions, and societal discourses attributed to the stigma of institutional aged care may be distinct from stigma associated with performing aged-care work in a noninstitutional setting (e.g., home care services). Health professionals may separate themselves from workers who deliver services in a facility based on the status loss associated with living/working in institutional aged care (Roth et al., 2016). Researchers have found that residents and workers who are linked with institutional aged-care facilities are often dehumanized and separated from the community, which leads to society recognizing them as a homogeneous outgroup (Gamliel & Hazan, 2006). Consequently, this may reinforce an “us vs. them” mentality between health professionals who work in the health care context and those working in institutional aged care.

A key difference between the quantitative and qualitative analyses was that our linguistic analysis found that health professionals interpret the social devaluation of aged care in relation to multiple targets (i.e., workers, work, institutions) and attitudes beyond just positive or negative valence. Participants conceptualized aged care in terms of affect (i.e., negative security), judgment (i.e., negative normality, negative tenacity), and appreciation (i.e., negative reaction, negative valuation; Martin & White, 2005). These attitude types can be understood with reference to the theoretical framework from the dirty work literature (Ashforth & Kreiner, 2014) which also helps explain the relationship between health professionals’ perceptions of occupational taint and social devaluation. Insecurity may be associated with physical taint as it describes an emotional response to performing work under dangerous conditions. Participants reported uneasiness associated with working in facilities during the pandemic in the qualitative responses. Given we did not find a direct effect of physical taint on intentions to work in aged-care institutions in the quantitative analysis, we suggest that these feelings of uneasiness may have been related to working in aged-care facilities, where providers were poorly equipped to manage the risks of exposure to COVID-19. The two categories of judgment (normality, tenacity) map onto social taint and moral taint, respectively, as it assesses people’s character based on social norms and moral sensibilities. Reaction refers to perceptions about the poor quality of occupational conditions in aged care. These conclusions suggest that evaluative language about aged care can be a resource that they use to construct their understanding of stigma.

### Limitations and Future Research

We acknowledge limitations include the scope of this study as it primarily explores factors that hinder willingness to work in aged care. Future studies can examine factors (i.e., intrinsic rewards, cultural expectations) that may promote willingness to work in aged care. Although a single question provided sufficient text to be able to identify reoccurring patterns in language, future studies could use additional questions that will allow for more data pertaining to other related targets that triangulate a fuller picture of the stigma of aged-care work. Our scope of analysis did not explicitly examine people receiving aged care as a target of stigmatizing discourses. Future research may explore how health professionals position themselves to stigmatizing discourses pertaining to clients in aged care. Additionally, this study did not involve examining how participants’ current/previous roles affected their responses. Future studies could test the interplay between stigma, health professionals’ work experiences (e.g., in or outside aged care, institutional or community), and work intentions.

We chose to frame the question for our linguistic analysis around COVID-19 because we recognized that the aged-care sector has been challenged and been the source of much public discourse in this context. For example, reporting of poor risk management of personal contagion risk to COVID-19 in aged care may have reinforced the undesirable qualities of aged-care work because it highlights anxiety of possibly spreading the virus to clients and families (Blanco-Donoso et al., 2021). Despite examining whether or not participants referenced the stigma of aged-care work in this context, we did not explicitly examine whether perceptions about aged care were *changed* by COVID-19 because it was beyond the scope of our research. Future research could examine whether attitudes about aged care have changed throughout COVID-19.

Another interesting future research direction is examining language underlying the stigma of working in aged care as it unfolds during the attraction, recruitment, and retention process. Researchers may identify linguistic patterns at different stages of the attraction/recruitment/retention processes, which may provide insight into how workers’ alignment/disalignment with social discourses can contribute to work outcomes at multiple levels (e.g., turnover, engagement, employee well-being, team-based care, patient experiences). Further research is needed to provide an exemplar of successful engagement, which can be used as a model for reframing stigmatizing discourses. Studies may consider observing intergroup communication in practice and/or analyzing naturally occurring conversations between these social groups to attain insight into these social processes.

### Implications for Theory and Policy

Our study provides theoretical insights and informs policymaking in three fundamental ways. First, we integrate the theoretical frameworks of the dirty work literature (Ashforth & Kreiner, 2014) with Stigma Theory (Link & Phelan, 2001; Yang et al., 2007) to extend the existing scope of research about the implications of the stigma of working in aged care on recruitment (Manchha et al., 2021). To date, researchers have predominantly examined the pairing between the nature of stigma associated with aged-care work (i.e., status, negative professional image, pay parity) and barriers toward recruiting aged-care workers (Chenoweth & Lapkin, 2018). This study is one of the first to conceptualize and test theory-based aspects of occupational stigma, such as the distinction between the relationship of the stigma of working in aged care on willingness to perform aged-care work and working in an institutional aged-care facility. Findings will help reframe policymaking and practices to respond to this formidable recruitment crisis with a nuanced focus on addressing the additional stigma emerging from the institutional aged-care context.

Second, we filled the void of research about the nature of aged-care work and all three aspects of dirty work. Our findings align with past research (Banks, 2018; Clarke & Ravenswood, 2019) that has conceptualized aged-care work as physically and socially tainted. We provide empirical evidence to argue aged-care work is also associated with moral taint, which has been previously overlooked in the literature. Thus, aged care is another “dirty” occupation that epitomizes a trifecta of taints. The challenge of recruitment could also be due to this additional taint that may further constrain potential workers from engaging in aged-care work based on moral sensibilities. We propose that our surfacing of perceptions that create moral taint have repercussions for regulatory systems within the aged-care sector and the redesign of accreditation/governance to minimize unethical behaviors. Thereby, system reforms may assist with reducing moral taint and increasing employee attraction to work in this field.

Third, this study demonstrates how the stigma of working in aged care affects affiliates of the aged-care workforce. Past research has focused on the implications of this stigma for aged-care workers (Manchha et al., 2021); however, we recognize the stigma of working in aged care may also constrain how health professionals engage with the aged-care sector. Thus, policymakers need to consider the role of stigma when designing guidelines for delivering multidisciplinary services within aged-care facilities (Davidson & Szanton, 2020).

Implications for addressing the impact of the stigma of working in aged care on recruitment relate to the media, public messaging, and training. Findings from our study suggest that the media is an influential communication channel for health professionals. For this reason, we recommend future research could look into how to establish good practices for media coverage. In the short term, solutions at an institutional level may include organizations and aged care providers using these linguistic findings when developing public messaging for attracting and recruiting health professionals. Specifically, the language used in job advertisements, promotional material, and educational resources may need to be refined to ensure it acknowledges alternative positions to enable health professionals the opportunity to challenge or reject societal discourses about the devaluation of aged care.

Furthermore, this study recognizes how training may provide opportunities for health professionals and management to develop a more expansive position about aged care (i.e., openness to alternative views). Current training (see McAllister, Ryan et al., 2020) involves working with health professionals during their undergraduate/in-service training to support more positive attitudes about aged care and their clients. We propose an essential part of training involves evaluating media messages, as a majority of participants reproduced discourses endorsed by the media. Through open discussions led by language-trained facilitators, health professionals can clarify or evaluate their position critically (e.g., “what makes you think that way?”) to establish their linguistic resources for presenting their own perspectives. Additionally, we propose language as a resource that can enable health professionals and the aged-care workforce to manage these devaluing discourses. Individuals with limited linguistic resources are more inclined to align with dominant societal discourses because they are less equipped to entertain alternative positions (Martin & White, 2005). Thus, interventions that focus on expanding individuals’ linguistic resources may offer a novel strategy for both managing this stigma and potentially challenging the projected position at a societal level.

## Conclusions

This mixed-methods study contributes to research about the interplay of stigma, aged care, and work intentions. Specifically, we investigated the relationship between health professionals’ perceptions of occupational taint, conditions associated with working in aged care, and willingness to work in aged care to better understand the implications of stigma on health professionals’ work intentions. Quantitative findings supported extant literature that positioned aged care as a “dirty” occupation. We identified that perceptions of taint do influence health professionals’ willingness to work in aged-care institutions when mediated by perceptions of social devaluation about aged care. Building on these results and Stigma Theory, we employed a linguistic analysis to examine how health professionals’ language choices construct their position toward the social devaluation of aged care. Findings revealed that health professionals aligned with negative societal discourses about aged care. Health professionals demonstrated they (re)produce the stigma reflected in the media. This study suggests understanding stigma and language choices may help us develop insightful strategies for overcoming aged-care workforce challenges.

## Supplementary Material

gnac002_suppl_Supplementary_Material

## Author Notes

1. Nine participants did not satisfy the requirements of our attention checks and were excluded from the quantitative data set. However, 168 participants’ (46% allied health, 27% nursing, 14% social assistance, and 13% doctor) written responses were included in the linguistic analysis as their written responses indicated that they had fully engaged with the open-ended questions.
2. We framed perceptions in terms of “most people in society tend to think …” in line with the perspective that stigma is internalized in relation to another person’s positioning (Link & Phelan, 2001).
3. The words “counter,” “pronounce,” “endorse,” “entertain,” and “attribute” are considered technical terms in the engagement system (Martin & White, 2005, p. 134), marked by italics in this article.
