# Supplementary material for: The Social Construction of Stigma in Aged-Care Work: Implications for Health Professionals’ Work Intentions
Source: Gerontologist. 2022 Jan 8;62(7):994–1005. doi: 10.1093/geront/gnac002 (PMC9372892; doi:10.1093/geront/gnac002)
Supplement: gnac002_suppl_Supplementary_Material [file gnac002_suppl_supplementary_material.docx]

*The Gerontologist* Online Supplementary Material: Manchha, A.V., Way, K.A., Tann, K., Thai, M. The social construction of stigma in aged-care work: Implications for health professionals’ work intentions.

**Supplementary S1: Cross-Profession Comparisons**

A one-way ANOVA was conducted to compare differences between professional roles (nurse, doctor, allied health and social assistance) on each of the variables of interest. There was only a significant difference in perceptions of poor occupational conditions between professional roles [*F*(3,155)=4.12, *p* = .008]. Post-hoc comparisons using the Hochberg’s GT2 test were carried out because we had unequal sample sizes. The only significant difference between professions was that allied health professionals perceived significantly worse poor occupational conditions (*M* = 5.23 , *SD* = 1.23) than doctors (*M* = 4.20, *SD* = 1.12), *F*(1, 92) = 11.37, *p* = .001. No other cross-profession differences emerged for any other variable, *F*s < 2.53, *p*s > .059.

Findings revealed a significant difference between doctors and allied health professionals’ perceptions of poor occupational conditions, in which allied health professionals reported poorer occupational conditions in aged-care work. We suggest this difference could be explained by allied health professionals experiencing poorer occupational conditions in aged care than in other sectors they had worked (e.g., limited access to staff & equipment deficits, Ostaszkiewicz et al., 2016), which may reinforce their negative perceptions. However, due to unequal sample sizes and low numbers in each condition (n= 20 doctors vs n= 74 allied health professionals), this finding should be interpreted with caution. Further examination is required to understand these differences.
